# Uterine Cancer: American Cancer Society Atlas of Clinical Oncology

**DOI:** 10.1038/sj.bjc.6602352

**Published:** 2005-01-25

**Authors:** L H Ellenson

**Affiliations:** 1 Weill Medical College of Cornell Universitym, New York, NY USA

This textbook is part of a series entitled the Atlas of Clinical Pathology from the American Cancer Society. As there are very few textbooks devoted to malignancies of the uterus, it is a welcome publication. Although it is limited in scope to uterine cancer, it is has considerable breadth. It includes topics such as epidemiology, molecular genetics, a thorough discussion of uterine cancer pathology, and clinical issues ranging from detection and screening to postsurgical management. Thus, it will appeal to an audience with a wide range of interests in uterine cancer.

Each chapter is written in a comprehensive manner appropriate for all, ranging from those with little background knowledge in uterine cancer to experts in the field. The chapters are organised in a logical order, which takes the reader on an educational journey that at its end leaves one with a complete and balanced understanding of all of the malignancies of the uterus. For example, discussion of the uncommon tumour types is left to the end and discussed separately in accordance with clinical relevance. Although the book is written by a number of authors, it has been carefully edited to alleviate overlap and provide continuity from chapter to chapter. In addition, the tables have all been formatted in the same manner giving the chapters a sense of conformity that allows the reader to focus on the information.

As a pathologist and molecular biologist, it was appreciated that both of these subjects, crucial to understanding the biology of uterine malignancies, were covered in appropriate detail. The chapters on pathology are well illustrated and emphasised the importance of diagnostic surgical pathology to the treatment of women with uterine cancer. The chapter on molecular pathogenesis may have been best titled ‘Molecular pathogenesis of endometrial carcinoma’ as it was limited to a discussion of this entity. Although less is known about the molecular genetic alterations in many of the other uterine malignancies, there is a body of literature for each tumour type. However, this is a minor point and does not detract from the chapter. This chapter will, in fact, be very helpful to the majority of readers and will give them a basic understanding of what is currently known about the molecular pathogenesis of endometrial carcinoma. The clinically oriented chapters are thorough and leave one with the distinct impression that everything you need to know is contained within. All clinically relevant issues are carefully discussed and presented in a balanced manner.

If the goal of this book was to provide a treatise on uterine malignancies that would be appropriate for students and experts alike, the goal has been achieved. It belongs on a shelf belonging to anyone with an interest in malignancies of the uterus.

